# Exploring multi-granularity balance strategy for class incremental learning via three-way granular computing

**DOI:** 10.1186/s40708-025-00255-0

**Published:** 2025-03-17

**Authors:** Yan Xian, Hong Yu, Ye Wang, Guoyin Wang

**Affiliations:** 1 Chongqing Key Laboratory of Computational Intelligence, Chongqing University of Posts and Telecommunications, No.2 Chongwen Road, Chongqing, 400065 China; 2 National Center for Applied Mathematics in Chongqing, Chongqing Normal University, No. 37 Middle University Road, Chongqing, 401331 China

**Keywords:** Class incremental learning, Episodic memory, Imbalance, Three-way granular computing, Contrastive learning

## Abstract

Class incremental learning (CIL) is a specific scenario in incremental learning. It aims to continuously learn new classes from the data stream, which suffers from the challenge of catastrophic forgetting. Inspired by the human hippocampus, the CIL method for replaying episodic memory offers a promising solution. However, the limited buffer budget restricts the number of old class samples that can be stored, resulting in an imbalance between new and old class samples during each incremental learning stage. This imbalance adversely affects the mitigation of catastrophic forgetting. Therefore, we propose a novel CIL method based on multi-granularity balance strategy (MGBCIL), which is inspired by the three-way granular computing in human problem-solving. In order to mitigate the adverse effects of imbalances on catastrophic forgetting at fine-, medium-, and coarse-grained levels during training, MGBCIL introduces specific strategies across the batch, task, and decision stages. Specifically, a weighted cross-entropy loss function with a smoothing factor is proposed for batch processing. In the process of task updating and classification decision, contrastive learning with different anchor point settings is employed to promote local and global separation between new and old classes. Additionally, the knowledge distillation technology is used to preserve knowledge of the old classes. Experimental evaluations on CIFAR-10 and CIFAR-100 datasets show that MGBCIL outperforms other methods in most incremental settings. Specifically, when storing 3 exemplars on CIFAR-10 with Base2 Inc2 setting, the average accuracy is improved by up to 9.59% and the forgetting rate is reduced by up to 25.45%.

## Introduction

The incremental learning [1–4] is also known as continual learning or lifelong learning, which means updating the model in a series of data stream. It is widely used in the fields of semantic segmentation [5], natural language processing [6], and object detection [7], etc. There are three primary scenarios in incremental learning: task incremental learning (TIL), domain incremental learning (DIL), and class incremental learning (CIL). In this paper, we focus on the CIL scenario without task identities, where the model learns from a data stream of new classes and can classify all observed classes at any time.

The CIL method is known to suffer from catastrophic forgetting, where the model struggles to recognize old classes after learning new ones, leading to poor generalization [8]. This phenomenon contrasts with the ability of humans to learn incrementally without forgetting old knowledge. The biological basis of human incremental learning ability is mainly derived from the neural replay mechanism of the hippocampus, which consolidates the memory storage in the neocortex by activating stored episodic memories [9, 10]. Inspired by the function of the hippocampus, the CIL methods based on episodic memory replay store a limited set of representative samples from old classes and replay them when learning new classes, thereby enhancing the memory of the old class [11]. In addition, methods based on non-episodic memory replay primarily integrate prior knowledge by using regularization terms [12] or dynamic expansion of network architectures [13], thus preserving old class knowledge to some extent. The researches has shown that episodic memory replay-based methods achieve superior performance than another [14, 15], as the replay of prior experiences is considered essential for stabilizing new memories [9].

**Fig. 1:**
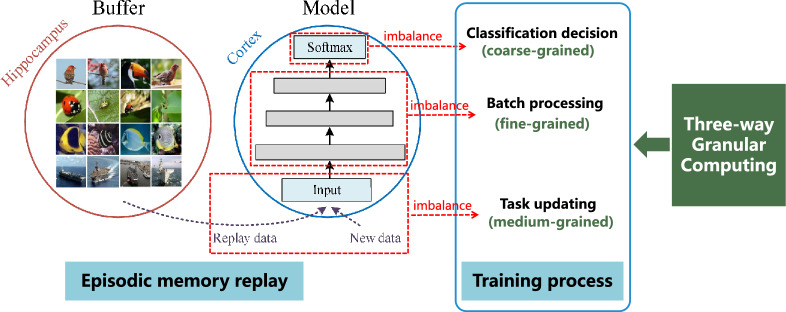
Schematic diagram of multi-granularity imbalance of episodic memory replay methods in the training process

To mitigate catastrophic forgetting, some episodic memory replay-based learning methods have been proposed. These methods stored representative exemplars from old classes as episodic memory [16–19]. Meanwhile, episodic memory replay can be naturally combined with knowledge distillation (KD) to incorporate previous knowledge from old model [11, 18, 20, 21]. However, although knowledge distillation can reduce forgetting to a certain degree, they still face challenges related to the imbalance of training samples between new and old classes. As illustrated in Fig. 1, episodic memory replay-based methods [22] are constrained by a limited buffer budget during incremental learning stages, allowing only a small number of old class exemplars to be stored. Consequently, the training process encounters a large number of samples from new classes compared to the relatively few old ones from old classes, resulting in an imbalance between new and old class samples. The class-specific weights are adversely affected by the imbalance, which produces a notable bias towards the new classes and ultimately leads to the forgetting of old classes.

The aforementioned issue of imbalance persists across different stages of the incremental training process, including batch processing, task updating, and classification decision. Specifically, the batch processing focuses on samples, the task updating combines both new and old class samples along with their labels, and the classification decision categorizes the labels. These three stages display a hierarchical structure at the data level, which is closely related to the hierarchical nature of human cognition. Zadeh [23] identified three basic concepts of human cognition: granulation, organization, and causation. Among them, the granular cognition mechanism is inspired by the human ability to granulate and reason with information. Yao [24] proposed the granular computing based on this mechanism, decomposing whole systems into multiple components and processing information through different granularity levels. The granular computing is conceptualized as a general theory for problem-solving, including two types of operators: one type deals with the transition from fine-grained to coarse-grained levels, and the other type handles the shift from coarse-grained to fine-grained levels. In the method of granular computing [25], three-way decision is an important method and has been widely used. Its basic concept is thinking in threes, which deals with a whole through three distinct but related parts [26, 27]. This corresponds to granular computing in threes, i.e., three-way granular computing [28]. In the last few years, we have witnessed the rapid development of three-way granular computing in multivariate time series forecasting [29], multi-view clustering [30], and multi-label classification [31]. Therefore, inspired by three-way granular computing, we map the batch processing, task updating, and classification decision stages of the incremental training process to three different granularity levels. The batch processing is at the fine-grained level as it handles the sample carefully, the task updating is at the medium-grained level by integrating samples and labels from both new and old classes, and the classification decision is regarded as the coarse-grained level because it categorizes labels for the entire task.

These methods are designed to solve the imbalance problem of class incremental learning [32–36]. They only address the imbalance between new and old class samples at the classification decision or task updating of training process, but they tend to ignore the imbalance that also exists at the batch processing. Therefore, we propose a novel CIL method based on multi-granularity balance strategy (MGBCIL), which is inspired by the three-way granular computing for human problem-solving. MGBCIL combines three granularities of the training process to mitigate the forgetting caused by the imbalance. Meanwhile, knowledge distillation is introduced to retain the knowledge of old classes and further mitigate forgetting. Specifically, at the fine-grained level of batch processing, we define a weighted cross-entropy loss with a smoothing factor to assign different weights to old and new classes’ samples, rather than the traditional cross-entropy loss that treats the loss calculation of each sample equally. In addition, during the medium-grained level of task updating and coarse-grained level of classification decision, we introduce the contrastive learning with different anchor settings to promote the local and global separation of new and old classes respectively.

To verify the effectiveness of the proposed MGBCIL method, we conduct extensive experiments on CIFAR-10 and CIFAR-100 datasets, showing superior performance compared to other methods. In particular, when the number of exemplars is 3 on CIFAR-10 with Base2 Inc2 setting, the average accuracy improves by up to 9.59% and the forgetting rate reduces by up to 25.45%. In conclusion, our main contributions are summarized as follows:

- A novel CIL method is proposed to achieve balance among the three granular levels within the training process, which is inspired by the three-way granular computing for human problem-solving.
- As far as we know, it is the first time that the weighted cross-entropy loss is designed to rebalance the samples of new and old classes in batch processing.
- We introduce contrastive learning with different anchor settings during the task updating and the classification decision, thereby promoting the local and global separation of new and old classes.

This paper is an extended version of an earlier conference paper [37]. We conduct a deeper analysis of the causes and processes of imbalance in incremental training, as shown in Fig. 1. Additionally, we expand all evaluations to multiple incremental settings and add new comparison methods to strengthen the experimental analysis. A notable finding is that MGBCIL performs better when there is a high degree of imbalance between the new and old class samples.

## Related work

The well-known problem of catastrophic forgetting is addressed in numerous CIL methods, which can be coarsely categorized into two main learning paradigms: non-episodic memory replay-based and episodic memory replay-based. In the first learning paradigm, these can be further subdivided into regularization-based and network architecture-based.

### Regularization-based Methods

The methods constraint the loss function related to the current classes, preventing the overwriting of previous knowledge with new information. MAS [38] adopted online and unsupervised strategies to gather importance measures, relying on the sensitivity of prediction results to parameter changes, thus effectively protect critical knowledge related to previous task will not be covered. PODNet [12] employed a spatial distillation loss to maintain representations within the model. By penalizing feature changes of input samples across old and new feature spaces, the parameter adjustment is indirectly affected. The method preserves the knowledge of old classes and mitigates forgetting during incremental learning. The significance of every parameter is approximated online via SI [39] based on its contribution to the total loss variation and the extent of its updates throughout the training process. Nevertheless, the regularization-based methods’ performance tend to be lower than that of the episodic memory replay-based methods [40].

### Network architecture-based methods

The methods extend models designed for previous classes to incorporate current classes by maintaining varying degrees of parameter isolation between the previous and current components. This isolation is typically achieved by adding new branches [41], [13] or splitting existing connections [42]. $H^2$ [43] explicitly optimizes a binary mask in a fixed network design, allocating specific neurones or parameters for each task while freezing masked areas for previous tasks. However, CPG [44] allow the network to dynamically expand when the network architecture’s capacity is not sufficient to effectively learn a new task. These methods are commonly applied in TIL scenarios with provided task identifiers. However, the parameter size increases with the number of seen tasks, posing challenges in CIL scenarios without task identifiers compared to episodic memory replay-based methods. In these scenarios, the methods either fail or perform poorly [40].

### Episodic memory replay-based methods

The methods mitigate catastrophic forgetting by flexibly transferring a limited set of exemplars from old classes, simulating the function of the hippocampus of the human brain to replay the episodic memory. The Coil [18], DER [17], and Foster [19] adopted a herd selection strategy, selecting representative exemplars of old classes based on distance histograms and storing them in episodic memory. GEM [16] stored the last *m* samples of representative exemplars from each task as episodic memory. ER [45] proposed reservoir sampling to manage exemplars within a fixed buffer budget, assuming that data streams follow an independently and identically distributed pattern. DRI [46] trained a generative model to supplement old training samples with generated data. Meanwhile, episodic memory replay can be naturally combined with knowledge distillation. Co2L [20] introduced the constrastive loss and preservation mechanism for robust representations against catastrophic forgetting. iCaRL [11] decoupled the learning of the classifier and feature representation, where the classifier was implemented using exemplars from the episodic memory and feature representation was derived through knowledge distillation [47]. To mitigate data imbalance with limite old training samples, Bic [32] introduced a bias correction method that used a linear model to adjust the distribution of output logits. IL2M [33] suggested a dual-memory approach that adjusted prediction scores by storing both statistical data and exemplars from old classes. LUCIR [34] used cosine normalization and hard negative mining to align features orientations between old and new models. FSCIL [48] showed that knowledge could be effectively retained by learning the topology of the manifold feature space, even when the manifold exhibits non-uniform and heterogeneous properties. SS-IL [35] employed separated softmax in the last layer and task-wise KD to reduce the impact of imbalance. Memo [49] identified that the shallow layers of a model exhibited greater generality, while the deeper layers were more task-specific. Consequently, it decoupled the intermediate backbone by proposing a modular design. PCR [36] proposed replacing anchor contrastive samples with corresponding proxies, which achieved a complementary balance between proxy-based and contrastive replay, thereby effectively mitigating challenges caused by class imbalance.

The replay-based methods usually address the imbalance between new and old class samples at medium- or coarse-grained levels, primarily focusing on classification decision or task updating during the training process. However, it is common to ignore the imbalance that occurres at the fine-grained level of the training process, such batch processing. To address this problem, we propose a multi-granularity balance strategy for the CIL method that includes three granularities to comprehensively mitigate imbalances across different stages of the training process.

## The proposed method

**Fig. 2:**
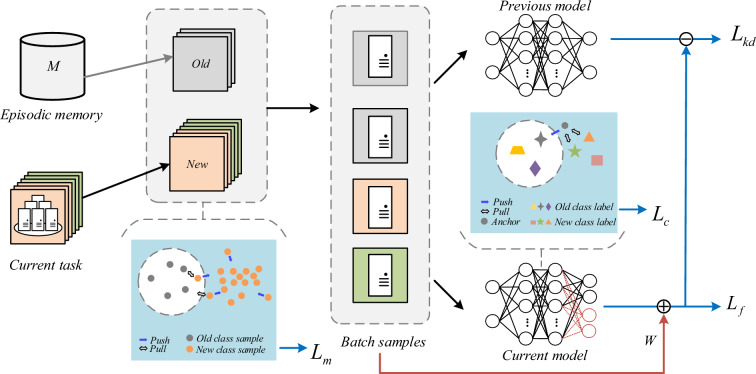
Overview of the proposed MGBCIL framework

In this section, we provide a detailed introduction to the proposed MGBCIL method and the overall framework of MGBCIL is illustrated in Fig. 2. To mitigate the classification bias caused by the imbalance between old and new class samples, we design the multi-granularity balance strategy inspired by the three-way granular computing. Specifically, the training process is divided into fine-, medium-, and coarse-grained stages for batch processing, task updating, and classification decision respectively. $L_{f}$ represents the proposed weighted cross-entropy loss, ensuring all classes are fully learned at fine-grained stage. $L_{m}$ treats the current task samples as anchors and others as negative samples, promoting local separation between old and new classes at medium-grained stage. Meanwhile, $L_{c}$ sets the old classes as anchors and new classes as negative samples, encouraging global separation between new and old classes at coarse-grained stage. The multi-granularity balance strategy helps mitigate the adverse impact of imbalances on the resolution of catastrophic forgetting. Additionally, $L_{kd}$ is used to distill knowledge from old to new models, further preventing forgetting.

**Table 1:** Summary on important notations

Notations	Meanings
$C_{i}$, $D_{i}$, $N_{C_{i}}$, *N*	The class, the datasets for class $C_{i}$, the number of samples for class $C_{i}$, the total number of samples in the current task
$C_{old}$, $C_{new}$, *k*, $\delta$	The set of old class, the set of new class, the number of old class, the number of new class
$M_{old}$, $M_{new}$	The episodic memory of old classes, episodic memory after cumulative updating
$\varvec{x}$, *y*, $N_b$	The sample in batch processing, the ground truth label of sample, the number of samples in batch processing
$L_{f}$, $L_{m}$, $L_{c}$, $L_{kd}$	Fined-grained loss, medium-grained loss, coarse-grained loss, knowledge distillation loss
$\text {NWs}(C_{i})$, $\varvec{W}$	The smoothed and normalized weight of class $C_{i}$, the weight vector of the current class
$\text {Enc}(\theta )$, $\text {Proj}(\phi )$, $\text {H}(\cdot )$, $\text {Aug}(\cdot )$	Encoder, projection function, augmentation function, label embedding
*s*	The smoothing parameter of fined-grained loss
*m*	Margin of separation between old and new classes in coarse-grained losses

### Notation and preliminary

The notation table is included in Table 1, and we shall explain its precise meaning when first used. Assuming that *n* classes are needed for incremental learning, a random sequence of classes $C_{i}\in \left\{ C_{1}, C_{2}, \cdots , C_{n}\right\}$ is created. The dataset for class $C_{i}$ is denoted as $D_{i}=\left\{ \left( \varvec{x}_{1}^{(i)}, y_{1}^{(i)}\right) ,\left( \varvec{x}_{2}^{(i)}, y_{2}^{(i)}\right) , \cdots ,\left( \varvec{x}_{N_{C_{i}}}^{(i)}, y_{N_{C_{i}}}^{(i)}\right) \right\}$, where each class $C_{i}$ includes $N_{C_{i}}$ samples. We use the Base$\Theta$ Inc$\delta$ incremental setup, where $\Theta$ classes are introduced for the initial training phase (0-phase), and $\delta$ classes are added in each subsequent phase. Formally, we suppose the model is trained on old classes $C_{old}=\left\{ C_{1}, C_{2}, \cdots , C_{k}\right\}$, retaining the episodic memory $M_{old}$ of these classes. There are $k+\delta$ classes with a total number of samples $N=N_k+N_\delta$ in current task. We aim to train a classifier based on the new classes $C_{new}=\left\{ C_{k+1}, \cdots , C_{k+\delta }\right\} (k+\delta \le n)$ and $M_{old}$, and then evaluate its classification performance across all observed classes $\left\{ C_{1}, C_{2}, \cdots , C_{k+\delta }\right\}$, updating the accumulated episodic memory $M_{new}$.

### Multi-garularity balance strategy

#### The fine-grained of batch processing

In batch processing at a fine-grained level, the cross-entropy loss is commonly utilized to train the multi-class classifiers for each sample $\varvec{x}$:

1 $$L_f=-\sum _{i=1}^{\vert k+\delta \vert } y_{i} \log p_{i}$$

where $y_i$ denotes the ground-truth label of $\varvec{x}$, and $p_i$ is the probability derived from the softmax of the corresponding class.

However, we find that there is a significant sample imbalance between old and new classes during each fine-grained batch training. The traditional cross-entropy loss treats each sample equally, ignoring this imbalance. Thus, we propose a weighted cross-entropy loss to solve the imbalance by assigning different weights to samples from different classes, ensuring that all classes are fully learned within the batch processing. The total occurrence times $\operatorname {Tol}(C_{i})$ and frequency $\operatorname {Freq}(C_{i})$ of class $C_{i}$ are calculated as:

2 $$\begin{aligned} \operatorname {Tol}\left( C_{i}\right) =\sum _{j=1}^{N_b} I_{\left\{ \varvec{x}_{j} \mid y_{j}=C_{i}\right\} }\left( \varvec{x}_{j}\right) , i \in \{1,2, \ldots , k+\delta \}, \end{aligned}$$

3 $$\begin{aligned} \operatorname {Freq}(C_{i})=\frac{\operatorname {Tol}(C_{i})}{N_b}, \end{aligned}$$

where $\varvec{x}$ represents the sample in batch processing, *y* indicates its corresponding label, and $N_b$ is the number of samples in batch processing. The indicator function *I* returns a value of 1 if $y_{j}=C_{i}$ for sample $\varvec{x}_{j}$, and 0 otherwise. Subsequently, the weighted value for each class $C_{i}$ is calculated as:

4 $$\begin{aligned} \text{ Wc } (C_{i})=\frac{1}{\operatorname {Freq}(C_{i})}. \end{aligned}$$

To mitigate the impact of extremely low or high frequencies, a smoothing parameter *s* within the range of [0, 1] is introduced. When $s=0$, the weight is directly assigned as the reciprocal of the actual class frequency. When $s>0$, a smoothing term is added to the frequency value to prevent the influence of extreme frequencies. Due to the addition of *s*, the denominator does not become excessively small when $\operatorname {Freq}(C_{i})$ is low, nor does it fluctuate significantly when $\operatorname {Freq}(C_{i})$ is high. This has the effect of smoothing the weights and avoiding extreme situations.

5 $$\begin{aligned} \begin{aligned} \text{ Ws } (C_{i})&=\frac{1}{\operatorname {Freq}(C_{i})+s}\\&= \text{ Wc } (C_{i})(\frac{1}{1+ \text{ Wc } (C_{i})\cdot s}). \end{aligned} \end{aligned}$$

To ensure that the weights are distributed in a reasonable range, the class weights $\text {Ws}(C_{i})$ in Eq.(5) is normalized to ensure that maximum weight is 1:

6 $$\begin{aligned} \text {NWs}(C_{i}) = \frac{\text {Ws}(C_{i})}{\max (\text {Ws}(C_{i}))}, i\in \{1,2,\ldots ,k+\delta \}, \end{aligned}$$

where $\max (\text {Ws}(C_{i}))$ is the max value of class weights in this batch, so that the vector of weight is represented as follow:

7 $$\begin{aligned} \varvec{W} =\left\{ \text {NWs}(C_{i}) \mid i \in \{1, 2, \ldots , k+\delta \}\right\} . \end{aligned}$$

The weighted cross-entropy loss of the fine-grained is defined by Eq.(8), which can pay more attention to a few classes by giving a higher weight to these classes during batch training, reducing the impact of sample imbalance on the anti-forgetting performance of the model. The strategy allows the model to adapt its focus adaptively based on data distribution, learning more distinguishable features from a limited number of samples effectively.

8 $$L_{f}=-{\varvec{W}}\sum _{i=1}^{\vert k+\delta \vert } y_{i} \log p_{i}.$$

#### The medium-grained of task updating

During the medium-grained of task updating, an imbalance persists between new and old class samples across the current and previous tasks. Therefore, we introduce the asymmetric supervised constrastive learning to promote the local separation of new and old classes [20]. In this setup, samples from the current task are used as anchors, while the samples from previous task stored in the episodic memory buffer are treated as negative samples.

In batch processing, there are $N=N_k+N_\delta$ samplels and $k+\delta$ classes of the current task. The data augmentation function $\operatorname {Aug}(\cdot )$ applies a random transformation, resulting in $a \ne b \Leftrightarrow \operatorname {Aug}_{a}(\cdot ) \ne \operatorname {Aug}_{b}(\cdot )$. The index set for the samples of the current task is designated as $2N=\operatorname {Aug}_{a}(N) \cup \operatorname {Aug}_{b}(N)$. Each sample $\varvec{x}$ within 2*N* is mapped to an embedding vector $\varvec{r}=\operatorname {Enc}\left( \varvec{x};\theta \right)$ through the encoder $\operatorname {Enc}(\theta )$, and then transformed into a projection vector $\varvec{z}=\operatorname {Proj}\left( \varvec{r}; \varphi \right)$ using the projection function $\operatorname {Proj}(\varphi )$. The asymmetric supervised constrastive loss of the medium-grained is defined as:

9 $$\begin{aligned} \begin{aligned} L_{m}=\sum _{i \in S} \frac{-1}{\left| \mathfrak {p}_{i}\right| } \sum _{j \in \mathfrak {p}_{i}} \log \left( \frac{\exp \left( \varvec{z}_{i} \cdot \varvec{z}_{j} / \tau \right) }{\sum _{t \ne i} \exp \left( \varvec{z}_{i} \cdot \varvec{z}_{t} / \tau \right) }\right) ,\\ \end{aligned} \end{aligned}$$

10 $$\begin{aligned} \mathfrak {p}_i =\{q \mid i\ne j \wedge y_i=y_j,\ j\in \{1,2,\ldots ,2N\}\}, \end{aligned}$$

where $\tau$ represents a temperature hyper-parameter, and the index set of the current task samples in the batch is signified as $S \subset \{1, 2, \ldots , 2N\}$. The index set $\mathfrak {p}_i$ corresponds to the positive samples related to the anchor.

#### The coarse-grained of classification decision

In the classification decision stage, there is a class imbalance between the new and old classes. In order to avoid the confusion that may be caused by class imbalance, we promote their global separation through a margin ranking loss [34]. The strategy focuses on the overall distribution and relationship of classes, and considers the global separation between the new and old classes at the coarse-grained level.

In particular, we attempt to separate all new classes from the ground-truth label of old classes by the margin *m*. The sample $\varvec{x}$ of old classes in $N_k$ is regarded as the anchor from the memory buffer. The ground-truth label embeddings of the old classes are used as positive, and the Top-*L* new classes with the highest response to $\varvec{x}$ are taken as hard negative samples, using their ground-truth label embeddings as negative. It is defined as:

11 $$\begin{aligned} L_{c}=\sum _{l=1}^{L} \max \left( 0, -\langle \operatorname {H}(\varvec{x}), \operatorname {Enc}\left( \varvec{x};\theta \right) \rangle +\left\langle \operatorname {H}^{l}(\varvec{x}), \operatorname {Enc}\left( \varvec{x};\theta \right) \right\rangle +m\right) , \end{aligned}$$

where *m* is the margin threshold, $\operatorname {Enc}\left( \varvec{x};\theta \right)$ is the feature encoding of sample $\varvec{x}$ extracted by the current model, $\operatorname {H}(\varvec{x})$ is the label embedding of old class sample $\varvec{x}$, and $\operatorname {H}^{l}(\varvec{x})$ is the label embedding of the new class that responds to the Top-*L* of the old class sample $\varvec{x}$, which is chosen as hard negatives.

### Update the model

In order to further mitigate catastrophic forgetting, we incorporate a distillation loss *L*_*kd*_ to preserve knowledge from previous classes. By aligning the output distributions of the current model with those of the previous model, the distillation loss ensures that essential information from earlier learning stages is retained. This mechanism effectively reduces the tendency of the model to overwrite old knowledge while accommodating new information, thereby enhancing the overall stability and performance of the incremental learning process. The distillation loss is defined as:

12 $$L_{k d}=-\sum_{i=1}^{k} \tau_{i}\left(p^{*}\right) \log \left(\tau_{i}(p)\right) ,$$

where the soft label p^*^ for sample x is generated by the previous model trained on the old classes, and the *p* is generated by the current model trained on the new and old classes. The rescaling function $\tau _{i}(v)=v_{i}^{1 / \Omega } / \sum _{j} v_{j}^{1 / \Omega }$ with $\Omega =2$ is used to increase the weights of smaller values.

The proposed MGBCIL method addresses the effects of imbalance on catastrophic forgetting by integrating fine-grained, medium-grained, and coarse-grained strategies. Additionally, the incorporation of the distillation technology ensures effective retention of old knowledge while accommodating the learning of new class, further mitigating the forgetting. Therefore, we combine the above losses into a comprehensive total loss function, which consists of four terms:

13 $$\begin{aligned} L=L_{f}+\lambda L_{kd}+L_{m}+ L_{c}, \end{aligned}$$

where $\lambda =\lambda _{\text{ base } } \sqrt{\left| C_{new}\right| /\left| C_{old}\right| }$, and $\lambda _\text{base}$ is a fixed constant for each dataset.

## Experiment

### Preparation

#### Datasets

We evaluate the proposed model using two widely recognized datasets: CIFAR-10 and CIFAR-100 [50], which contain 10 and 100 classes, respectively. Each dataset comprises a total of 60,000 color images, with a resolution of $32 \times 32$ pixels per image. In the experiments on these datasets, 40,000 images are used for training, while 10,000 images are allocated for validation and another 10,000 are set aside for testing. To analyze the impact of memory constraints on incremental learning, we evaluate the model under four different exemplar storage settings: 3, 5, 8 and 10 exemplars per class.

#### Comparison method

Eight methods are compared with our method: Finetune [51] updated the model parameters without storing episodic memory. Several CIL methods were utilized the episodic memory replay, such as GEM [16], iCaRL [11], Bic [32], WA [52], Coil [18], Memo [49] and PCR [36]. These methods are described in detail as follows:

1. Finetune: The method fine-tuned the model parameters based on new classes without adding any episodic memory from old classes.
2. GEM: The method projected the gradient to the closest gradient satisfying the constraints, achieving a balance between learning new classes and retaining old knowledge.
3. iCaRL: The method extended knowledge distillation regularization by incorporating exemplar sets to retain previous knowledge. Additionally, it eliminated the fully connected layer and utilized nearest class mean (NCM) for classification during inference.
4. Bic: The method addressed the imbalance between new and old classes by introducing a dynamic balancing term. Furthermore, it proposed an additional rectification layer to adjust predictions. This layer was fine-tuned using a separate validation set split from the exemplar set to optimize performance.
5. WA: The method improved incremental learning by normalizing classifier weights after each optimization step. Additionally, it employed weight clipping to ensure that the predicted probabilities were proportional to the classifier’s weights.
6. Coil: The method introduced a bidirectional distillation framework through co-transport, leveraging the semantic relationships between new and old models to enhance incremental learning.
7. Memo: The method proposed decoupling the intermediate backbone and expanding the deeper layers to improve the model’s capacity for task-specific knowledge retention.
8. PCR: The method replaced the contrastive samples of anchor points with corresponding proxies, effectively solving the problem of imbalance.

#### Evaluation metrics

The Top-1 accuracy after the *b*-th phase is represented as $A_{b}$. Several evaluation metrics are selected to measure the performance in the public datasets [53], [2], including average accuracy $\bar{A}$, last phase accuracy $A_{B}$, and forgetting rate *F*.

1. Average accuracy ($\bar{A}=\frac{1}{B} \sum _{b=1}^{B} A_{b}$): This metric calculates the average accuracy across the initial and all incremental phases under various incremental settings. It provides a holistic view of the method’s performance throughout the entire incremental learning process and helps assess its stability in adapting to new tasks while retaining previously learned knowledge.
2. Last phase accuracy (*A*_*B*_): This metric represents the accuracy achieved at the end of the last incremental phase. It is a critical indicator for evaluating the ultimate performance of the model after learning all tasks, reflecting its capacity to retain knowledge from both the initial and incremental phases.
3. Forgetting rate ($F=(A_{B}-A_{0})/{A_{B}}$): This metric measures the percentage change in accuracy between the initial phase and the last incremental phase. A higher forgetting rate indicates greater loss of knowledge from earlier tasks, while a lower rate suggests better retention. It is particularly useful for assessing the robustness of methods in mitigating catastrophic forgetting during the incremental learning process.

#### Training process

In the training procedure, we implement the methods using PyTorch on a GeForce RTX 2080 Ti GPU, and some comparison methods based on the PyCIL toolbox  [54]. The classes within the datasets are arranged in a fixed random order, and the performance of model is evaluated on all observed classes after each phase. For the CIFAR-10 and CIFAR-100 datasets, the number of exemplars (numExe) are set to $\left\{ 3, 5, 8, 10 \right\}$. ResNet-18 is adopted as the encoder and backbone network for all methods. For MGBCIL, the projection function is an MLP with a hidden layer activated by ReLU. The initial learning rate is set to 0.01 and reduced by a factor of 10 after 80 and 120 epochs, with training spanning a total of 160 epochs. The hyper-parameters are configured as follows: $\lambda _\text{base}=5$, $\tau =0.07$, while the number of layers *L* is set to 1 for CIFAR-10 and 2 for CIFAR-100.

**Fig. 3:**
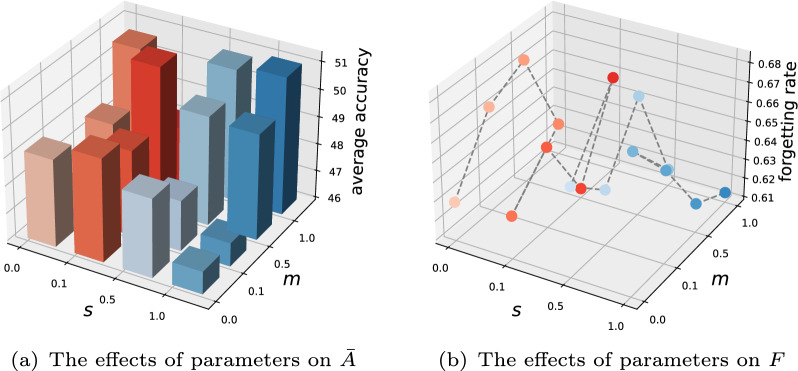
Parameter studies of MGBCIL on CIFAR-10 with Base2 Inc1 and numExe=3

### Parameter study

To enhance the stability of fine-grained loss, a smoothing parameter *s* is introduced to prevent extreme frequency impacts. Simultaneously, a margin threshold *m* is incorporated into coarse-grained loss to enforce a clear separation between new and old classes. This experiment explores the effects of these parameters on the incremental learning performance of MGBCIL. Specifically, we report the average accuracy $\bar{A}$ and forgetting rate *F* on the CIFAR-10 dataset under the Base2 Inc1 incremental learning setting, where the values of *s* and *m* are adjusted within the range of $\left\{ 0.0, 0.1, 0.5, 1.0 \right\}$.

As illustrated in Fig. 3(a) and (b), which depict the average accuracy and forgetting rate, respectively. MGBCIL achieves its optimal performance when $s=0.1$ and $m=0.5$. These parameter values ensure a balanced trade-off between two objectives: reducing the impact of extreme frequency in the fine-grained loss and maintaining effective separation between new and old classes in the coarse-grained loss. Based on these findings, we adopt $s=0.1$ and $m=0.5$ as the default parameter settings for all subsequent experiments. This parameter selection not only maximizes the overall effectiveness of MGBCIL but also provides validation for the reasonableness of its design.

### Comparison with existing work

In this section, we conduct comprehensive experiments to validate the effectiveness of MGBCIL in mitigating catastrophic forgetting and incremental classification. The proposed MGBCIL is compared with other methods on the CIFAR-10 and CIFAR-100 datasets. To further demonstrate the versatility of MGBCIL, we evaluate its performance under different episodic memory buffer budget settings, which is a crucial factor in incremental learning as it directly impacts the model’s ability to retain knowledge from old classes.

**Fig. 4:**
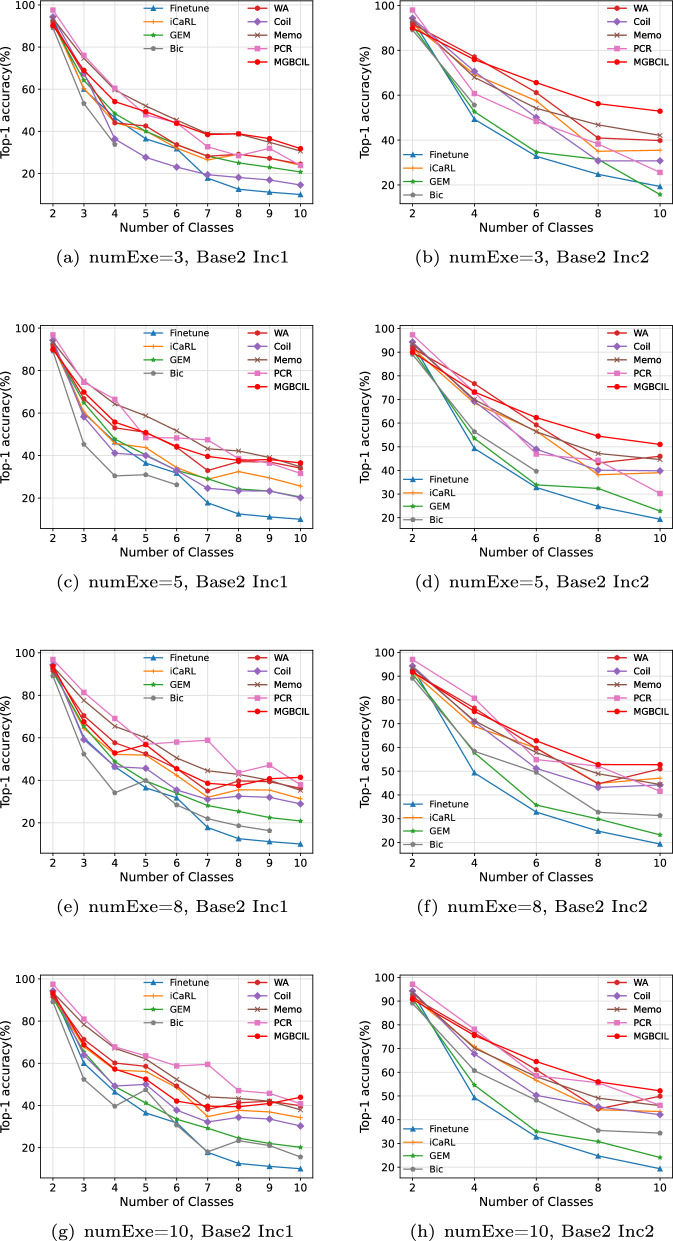
Top-1 accuracy variation on CIFAR-10

**Fig. 5:**
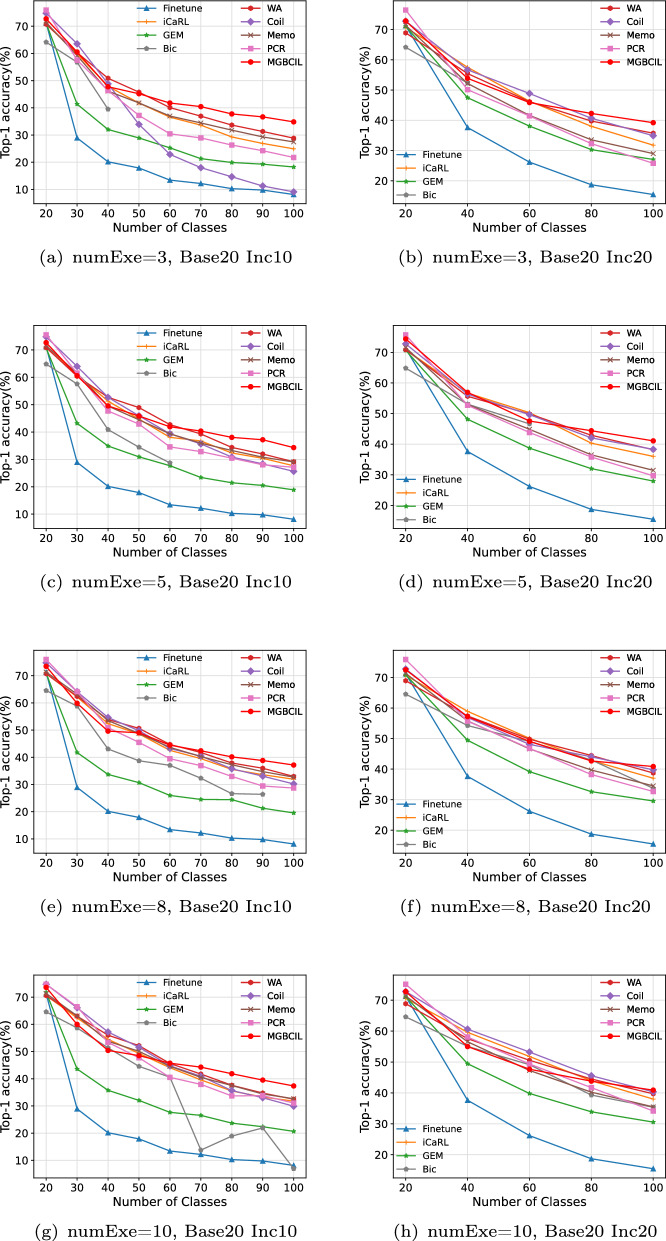
Top-1 accuracy variation on CIFAR-100

The advantage of the proposed MGBCIL method during the incremental learning process is clearly shown in Figs. 4 and 5, which displays the changes in Top-1 accuracy at each incremental phase. Specifically, as depicted in Figs. 4(a)-(e) and 5(a)-(e), the Bic method disrupts the incremental learning process when the numExe is to 8 or less. To ensure a fair comparison, we only evaluate the incremental classification results of the Bic method during the phases when the numExe exceeds 8, as shown in Figs. 4(g)-(h) and 5(g)-(h). The results of these two figures indicate a gradual decrease in classification accuracy for all methods as new classes are learned incrementally, which aligns with the phenomenon of catastrophic forgetting. This progressive decrease in accuracy reflects the difficulty of retaining previously learned knowledge while incorporating new information. Although it is impossible to completely eliminate forgetting in incremental learning, the findings highlight that the effects of forgetting can be mitigated to a certain extent.

As shown in Figs. 4 and 5, when numExe is set variably within the range of 3 to 10, the forgetting phenomenon of MGBCIL is relatively stable without any sudden decreases or increases. Although the proposed MGBCIL method performs slightly inferior to the Coil and PCR methods in the early phase, it is completely superior to other comparison methods in the later phase of the incremental learning, and its performance is optimal on the final phase of incremental learning. In conclusion, these results demonstrate the superior ability of MGBCIL to retain old knowledge, highlighting its effectiveness in mitigating catastrophic forgetting. The findings provide strong evidence of its excellent performance across various incremental settings. This further underscores the importance of strategies such as multi-granularity balance and knowledge distillation in reducing the impact of forgetting.

In addition, we report several key performance metrics, including the average accuracy $\bar{A}$ across all incremental phases, the final phase accuracy $A_{B}$, and the percentage increase or decrease of the forgetting rate *F*. In these evaluations, the symbol $\uparrow$ indicates that larger values are better, while the symbol $\downarrow$ signifies that smaller values are better. For clarity, the best results are highlighted in bold, and the second-best results are shown in underline. As shown in Tables 2, 3, 4,5, MGBCIL performs better than other methods in the experimental settings of CIFAR-10 with Base2 Inc2, CIFAR-100 with Base20 Inc10, and CIFAR-100 with Base20 Inc20. In particular, the last phase accuracy $A_{B}$ and forgetting rate *F* are almost the best perform. For example, when numExe=3 in Table 3, the average accuracy is improved by up to 9.59% and the forgetting rate is reduced by up to 25.45%. These findings confirm the robustness of MGBCIL across various memory settings, further validating its potential for incremental learning tasks.

The multi-granularity balance strategy in MGBCIL shows limited effectiveness under a low degree of imbalance between the old and new class samples. As shown in the experimental results in Table 2 that compared with Memo and PCR methods, the average accuracy of MGBCIL does not achieve optimal performance when using the Base2 Inc1 setting on the CIFAR-10 dataset. This phenomenon can be attributed to the relatively small number of new class samples in the incremental setting of Base2 Inc1, where the initial phase contains two classes and only one new class is added in each subsequent incremental phase. Consequently, during the three stages of the incremental training process (batch processing, task updating, and classification decision), there is a low degree of imbalance between the old class samples replayed from episodic memory and the new class samples introduced in the current task. In such cases, the advantage of the multi-granularity balance strategy has not been fully utilized, which is designed to address the imbalance problem between old and new class samples. As a result, the performance of the MGBCIL method is limited in this specific setting to some extent.

**Table 2:** The average accuracy $\bar{A} (\uparrow )$, last phase accuracy $A_{B} (\uparrow )$, forgetting rate $F (\downarrow )$ of methods on CIFAR-10 with Base2 Inc1

Dataset	NumExe	Indicator	Finetune [51]	iCaRL [11]	GEM [16]	Bic [32]	WA [52]	Coil [18]	Memo [49]	FCR [36]	MGBCIL
CIFAR-10	3	$\bar{A}$	35.51	41.68	41.58	-	43.11	35.33	**52.03**	49.18	50.21
		$A_{B}$	10.00	23.90	20.71	-	24.46	14.52	30.62	23.74	**31.78**
		*F*	0.89	0.74	0.77	-	0.73	0.85	0.67	0.76	**0.65**
	5	$\bar{A}$	35.51	43.60	41.55	-	49.71	39.78	**55.82**	54.28	51.39
		$A_{B}$	10.00	25.61	20.47	-	34.08	20.17	34.70	31.62	**36.53**
		*F*	0.89	0.72	0.78	-	0.63	0.79	0.63	0.67	**0.59**
	8	$\bar{A}$	35.51	48.52	41.73	-	51.96	45.03	56.62	**61.11**	52.71
		$A_{B}$	10.00	31.30	20.83	-	36.19	28.89	35.29	37.88	**41.37**
		*F*	0.89	0.66	0.77	-	0.61	0.69	0.62	0.61	**0.56**
	10	$\bar{A}$	35.51	51.60	41.75	37.45	54.68	47.28	57.86	**62.39**	53.08
		$A_{B}$	10.00	34.22	20.16	15.56	39.88	30.27	37.95	40.81	**43.87**
		*F*	0.89	0.63	0.78	0.83	0.57	0.68	0.59	0.58	**0.53**

**Table 3:** The average accuracy $\bar{A} (\uparrow )$, last phase accuracy $A_{B} (\uparrow )$, forgetting rate $F (\downarrow )$ of methods on CIFAR-10 with Base2 Inc2

Dataset	NumExe	Indicator	Finetune [51]	iCaRL [11]	GEM [16]	Bic [32]	WA [52]	Coil [18]	Memo [49]	FCR [36]	MGBCIL
CIFAR-10	3	$\bar{A}$	43.97	57.80	45.10	-	62.15	55.30	60.92	54.18	**68.11**
		$A_{B}$	19.34	35.49	15.75	-	39.80	30.77	42.09	25.59	**52.89**
		*F*	0.79	0.61	0.83	-	0.57	0.67	0.55	0.74	**0.41**
	5	$\bar{A}$	43.97	58.79	46.71	-	63.35	58.49	62.32	58.36	**66.20**
		$A_{B}$	19.34	39.02	22.81	-	45.99	39.83	44.60	30.23	**50.99**
		*F*	0.79	0.57	0.75	-	0.50	0.58	0.52	0.69	**0.43**
	8	$\bar{A}$	43.97	62.44	47.49	52.20	64.71	60.72	63.18	65.25	**67.05**
		$A_{B}$	19.34	47.07	23.19	31.30	50.94	44.21	44.39	41.55	**52.72**
		*F*	0.79	0.49	0.75	0.65	0.44	0.53	0.53	0.57	**0.43**
	10	$\bar{A}$	43.97	61.34	47.10	53.57	64.76	59.99	63.38	67.08	**67.79**
		$A_{B}$	19.34	43.36	24.08	34.36	49.89	42.13	45.93	46.07	**52.17**
		*F*	0.79	0.53	0.74	0.61	0.46	0.55	0.51	0.53	**0.43**

**Table 4:** The average accuracy $\bar{A} (\uparrow )$, last phase accuracy $A_{B} (\uparrow )$, forgetting rate $F (\downarrow )$ of methods on CIFAR-100 with Base20 Inc10

Dataset	NumExe	Indicator	Finetune [51]	iCaRL [11]	GEM [16]	Bic [32]	WA [52]	Coil [18]	Memo [49]	FCR [36]	MGBCIL
CIFAR-100	3	$\bar{A}$	21.33	41.29	30.82	-	44.28	32.99	42.06	38.75	**46.41**
		$A_{B}$	8.12	24.92	18.24	-	28.83	9.09	27.47	21.74	**34.83**
		*F*	0.89	0.65	0.74	-	0.59	0.88	0.62	0.71	**0.52**
	5	$\bar{A}$	21.33	43.68	32.41	-	45.60	44.15	43.94	42.3	**46.73**
		$A_{B}$	8.12	27.66	18.87	-	29.13	25.64	29.06	27.13	**34.31**
		*F*	0.89	0.61	0.73	-	0.59	0.66	0.59	0.64	**0.53**
	8	$\bar{A}$	21.33	46.34	32.53	-	47.89	47.33	47.27	44.89	**48.33**
		$A_{B}$	8.12	31.93	19.54	-	32.96	30.20	32.69	28.69	**37.15**
		*F*	0.89	0.55	0.72	-	0.53	0.60	0.54	0.62	**0.49**
	10	$\bar{A}$	21.33	46.78	33.71	35.70	48.22	48.21	47.56	46.58	**49.03**
		$A_{B}$	8.12	31.78	20.65	6.91	32.46	29.9	32.66	31.06	**37.35**
		*F*	0.89	0.55	0.71	0.89	0.54	0.60	0.54	0.58	**0.49**

**Table 5:** The average accuracy $\bar{A} (\uparrow )$, last phase accuracy $A_{B} (\uparrow )$, forgetting rate $F (\downarrow )$ of methods on CIFAR-100 with Base20 Inc20

Dataset	NumExe	Indicator	Finetune [51]	iCaRL [11]	GEM [16]	Bic [32]	WA [52]	Coil [18]	Memo [49]	FCR [36]	MGBCIL
CIFAR-100	3	$\bar{A}$	33.84	48.88	42.81	-	49.20	50.79	45.55	45.22	**50.82**
		$A_{B}$	15.47	31.78	27.06	-	35.74	34.91	29.00	25.80	**39.21**
		*F*	0.78	0.55	0.62	-	0.48	0.52	0.59	0.66	**0.46**
	5	$\bar{A}$	33.84	50.85	43.51	-	51.51	51.84	47.47	47.55	**52.88**
		$A_{B}$	15.47	36.02	27.95	-	38.20	38.32	31.47	29.68	**41.09**
		*F*	0.78	0.49	0.60	-	0.46	0.47	0.56	0.61	**0.45**
	8	$\bar{A}$	33.84	51.92	44.34	48.92	51.87	52.32	49.60	49.86	**52.47**
		$A_{B}$	15.47	37.02	29.56	33.71	38.73	39.77	34.51	32.66	**40.85**
		*F*	0.78	0.48	0.58	0.48	**0.44**	0.45	0.52	0.57	**0.44**
	10	$\bar{A}$	33.84	52.78	44.97	48.69	52.23	**54.51**	50.18	51.69	52.03
		$A_{B}$	15.47	38.00	30.55	35.35	39.71	40.35	35.48	34.15	**40.90**
		*F*	0.78	0.46	0.57	0.45	**0.42**	0.45	0.50	0.55	0.44

### Ablation experiment

The proposed MGBCIL method incorporates four components of loss functions, each designed to address specific challenges in incremental learning: $L_{f}$ rebalances the samples of new and old classes during batch processing to mitigate fine-grained imbalances. $L_{m}$ ensures the local separation during task updating, and $L_{c}$ promotes the global separation in classification decisions. $L_{kd}$ retains knowledge from old classes via distillation. To verify the benefits of each loss function in MGBCIL, we conduct the ablation study on the CIFAR-10 dataset with Base2 Inc1 and numExe=3 using several combinations.

**Fig. 6:**
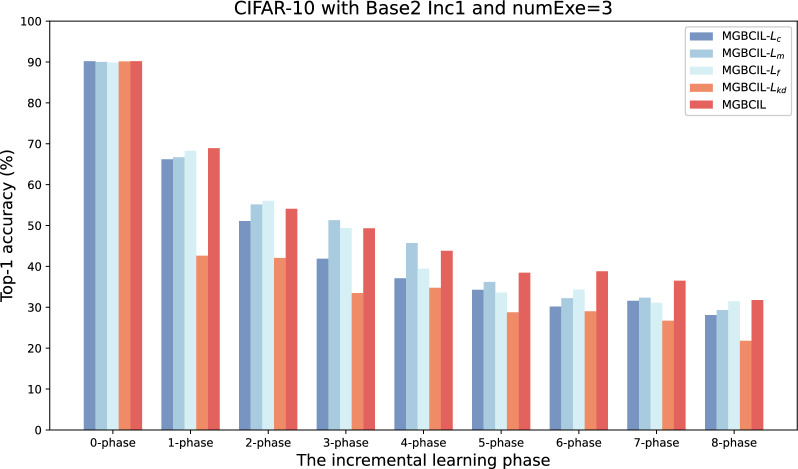
Top-1 accuracy for different components of MGBCIL in all incremental phases on CIFAR-10 with Base2 Inc1 and numExe=3

**Fig. 7:**
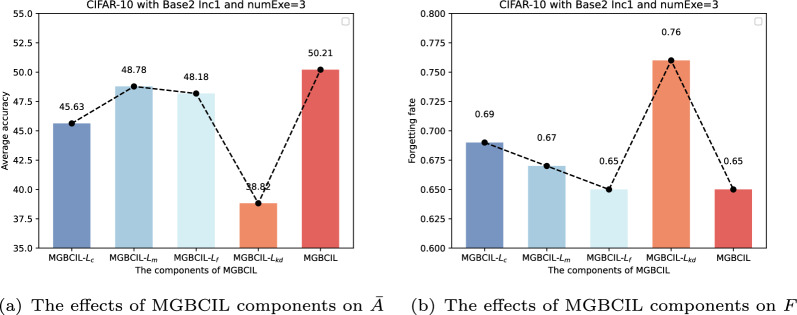
Average accuracy and forgetting rate for different components of MGBCIL on CIFAR-10 with Base2 Inc1 and numExe=3

As shown in Fig. 6, the experimental results show that MGBCIL with four loss components significantly outperforms other methods in classification accuracy at most phases of the entire incremental training process, especially from phase 5 to phase 8, where MGBCIL achieves the best results. Meanwhile, we find that MGBCIL has the highest average accuracy and the lowest forgetting rate in Fig. 7. These observations suggest that each loss function plays a crucial role in addressing the impact of the imbalance between new and old class samples, while also effectively mitigating the forgetting of previous knowledge. These findings highlight the necessity of four loss functions in the MGBCIL framework and emphasize their importance in incremental learning.

## Conclusion

In this paper, we propose a novel CIL method to mitigate forgetting caused by imbalance through the multi-granularity balance strategy, which is inspired by the three-way granular computing for human problem-solving. Specifically, we propose a weighted cross-entropy loss for rebalancing batch processing, and introduce the contrastive learning with different anchor settings in the task updating and the classification decision to promote the local and global separation of new and old classes. Additionally, the distillation loss also preserves previous knowledge to further mitigate catastrophic forgetting. According to experimental results, the proposed MGBCIL significantly outperforms the compared methods in various incremental learning settings, including classification accuracy and forgetting rate. These results verify the effectiveness of the MGBCIL in mitigating catastrophic forgetting.

According to the experimental results, MGBCIL does not get the highest average accuracy on the CIFAR-10 dataset with Base2 Inc1 incremental setting. This can be attributed to the fact that the number of new class samples is relatively small in the Base2 Inc1 setting, which reduces the degree of imbalance between new and old class samples and weakens the role of the multi-granularity balancing strategy. In future work, we aim to further explore an optimized multi-granularity balance strategy that can maintain efficient performance even in low degree of imbalance scenarios, or explore other strategies that operate effectively under various imbalance conditions.

## Data Availability

The CIFAR-10 and CIFAR-100 datasets analyzed for this study can be found in Alex Krizhevsky’s home page (https://www.cs.toronto.edu/~kriz/cifar.html).

## Code availability

The code is available at https://github.com/yuhongcqupt/MGBCIL

## References

[1] Zhou D, Wang Q, Qi Z et al (2024) Class-incremental learning: a survey. IEEE Trans Pattern Anal Machine Intell 46(12):9851–9873 10.1109/TPAMI.2024.3429383 39012754

[2] Wang L, Zhang X, Su H et al (2024) A comprehensive survey of continual learning: theory, method and application. IEEE Trans Pattern Anal Machine Intell 46(8):5362–5383 10.1109/TPAMI.2024.3367329 38407999

[3] Simon C, Koniusz P, Harandi M (2021) On learning the geodesic path for incremental learning. In: Proceedings of the IEEE/CVF Conference on Computer Vision and Pattern Recognition (CVPR), pp. 1591– 1600. IEEE, Nashville, TN, USA

[4] Zhou D, Sun H, Ning J, et al ( 2024) Continual learning with pre-trained models: A survey. In: Proceedings of the International Joint Conference on Artificial Intelligence (IJCAI), pp. 8363– 8371. Morgan Kaufmann, Jeju Island, South Korea

[5] Cermelli F, Mancini M, Bulo S, et al ( 2020) Modeling the background for incremental learning in semantic segmentation. In: Proceedings of the IEEE/CVF Conference on Computer Vision and Pattern Recognition (CVPR), pp. 9230– 9239. IEEE, Seattle, WA, USA

[6] Huang F, Zhang Y, Chen J, et al( 2021) Continual learning for text classification with information disentanglement based regularization. In: Proceedings of the Conference of the North American Chapter of the Association for Computational Linguistics: Human Language Technologies (NAACL-HLT), pp. 2736– 2746. Association for Computational Linguistics, Mexico City, Mexico

[7] Feng T, Wang M, Yuan H( 2022) Overcoming catastrophic forgetting in incremental object detection via elastic response distillation. In: Proceedings of the IEEE/CVF Conference on Computer Vision and Pattern Recognition (CVPR), pp. 9417– 9426. IEEE, New Orleans, LA, USA

[8] McCloskey M, Cohen NJ ( 1989) Catastrophic interference in connectionist networks: The sequential learning problem. In: Psychology of Learning and Motivation vol. 24, pp. 109– 165. Elsevier, Amsterdam, Netherlands

[9] Wilson MA, McNaughton BL (1994) Reactivation of hippocampal ensemble memories during sleep. Science 265(5172):676–679 8036517 10.1126/science.8036517

[10] Joo H, Frank L (2018) The hippocampal sharp wave-ripple in memory retrieval for immediate use and consolidation. Nat Rev Neurosci 19(12):744–757 30356103 10.1038/s41583-018-0077-1 PMC6794196

[11] Rebuffi SA, Kolesnikov A, Sperl G, Lampert CH ( 2017) Icarl: Incremental classifier and representation learning. In: Proceedings of the IEEE/CVF Conference on Computer Vision and Pattern Recognition (CVPR), pp. 5533– 5542. IEEE, Honolulu, HI, USA

[12] Arthur D, Matthieu C, Charles O, et al ( 2020) Podnet: Pooled outputs distillation for small-tasks incremental learning. In: Proceedings of the European Conference on Computer Vision (ECCV), vol. 12365, pp. 86– 102. Springer, Glasgow, United Kingdom

[13] Wang Z, Liu Y, Ji T et al (2023) Rehearsal-free continual language learning via efficient parameter isolation. Toronto, Canada, Association for Computational Linguistics

[14] Lomonaco V, Pellegrini L, Rodríguez P et al (2022) CVPR 2020 continual learning in computer vision competition: approaches, results, current challenges and future directions. Artif Intell 303:103635

[15] Mai Z, Li R, Jeong J et al (2022) Online continual learning in image classification: an empirical survey. Neurocomputing 469:28–51

[16] Lopez-Paz D, Ranzato MA ( 2017) Gradient episodic memory for continual learning. In: Proceedings of the 31st International Conference on Neural Information Processing Systems (NeurIPS), vol. 34, pp. 6467– 6476. MIT Press, Long Beach, CA, USA

[17] Yan S, Xie J, He X ( 2021) Der: Dynamically expandable representation for class incremental learning. In: Proceedings of the IEEE/CVF Conference on Computer Vision and Pattern Recognition (CVPR), pp. 3014– 3023. IEEE, Nashville, TN, USA

[18] Zhou DW, Ye HJ, Zhan DC ( 2021) Co-transport for class-incremental learning. In: Proceedings of the 29th ACM International Conference on Multimedia (ACM MM), pp. 1645– 1654. ACM, Chengdu, China

[19] Wang FY, Zhou DW, Ye HJ et al (2022) Foster: Feature boosting and compression for class-incremental learning. Springer, Tel Aviv, Israel

[20] Cha H, Lee J, Shin J (2021) l: Contrastive continual learning. IEEE, Montreal, QC, Canada

[21] Castro F, Marín-Jiménez M, Guil N et al (2018) End-to-end incremental learning. Springer, Munich, Germany

[22] Van de Ven G, Siegelmann H, Tolias A (2020) Brain-inspired replay for continual learning with artificial neural networks. Nature Commun 11(1):4069 32792531 10.1038/s41467-020-17866-2 PMC7426273

[23] Zadeh LA (1997) Toward a theory of fuzzy information granulation and its centrality in human reasoning and fuzzy logic. Fuzzy Sets Syst 90(2):111–127

[24] Yao Y (2004) A partition model of granular computing. Springer, Berlin, Heidelberg

[25] Wang G, Xu J (2014) Granular computing with multiple granular layers for brain big data processing. Brain Inf 1:1–10 10.1007/s40708-014-0001-z PMC4883151 27747523

[26] Yao Y (2020) Tri-level thinking: models of three-way decision. Int J Machine Learn Cyber 11(5):947–959

[27] Yao Y (2016) Three-way decisions and cognitive computing. Cognit Comput 8(4):543–554

[28] Yao Y (2018) Three-way decision and granular computing. Int J Appr Reason 103:107–123

[29] Yu H, Wang Z, Xie Y et al (2024) A multi-granularity hierarchical network for long- and short-term forecasting on multivariate time series data. Appl Soft Comput 157:111537

[30] Yu H, Wang X, Wang G et al (2020) An active three-way clustering method via low-rank matrices for multi-view data. Inf Sci 507:823–839

[31] Zhang Y, Zhao T, Miao D et al (2025) Three-way multi-label classification: a review, a framework, and new challenges. Appl Soft Comput 171:112757

[32] Wu Y, Chen Y, Wang L et al (2019) Large scale incremental learning. IEEE, Long Beach, CA, USA

[33] Belouadah E, Popescu A (2019) Il2m: Class incremental learning with dual memory. IEEE, Seoul, Korea

[34] Hou S, Pan X, Loy CC et al (2019) Learning a unified classifier incrementally via rebalancing. IEEE, Long Beach, CA, USA

[35] Ahn H, Kwak J, Lim S et al (2021) Ss-il: Separated softmax for incremental learning. IEEE, Montreal, QC, Canada

[36] Lin H, Zhang B, Feng S et al (2023) Pcr: Proxy-based contrastive replay for online class-incremental continual learning. IEEE, Vancouver, BC, Canada

[37] Xian Y, Yu H, Wang Y et al (2024) A novel class incremental learning method via multi-granularity balance inspired by human granular cognition mechanism. Springer, Bangkok, Thailand

[38] Aljundi R, Babiloni F, Elhoseiny M et al (2018) Memory aware synapses: Learning what (not) to forget. Springer, Munich, Germany

[39] Zenke F, Poole B, Ganguli S ( 2017) Continual learning through synaptic intelligence. In: Proceedings of the 34th International Conference on Machine Learning (ICML), vol. 70, pp. 3987– 3995. PMLR, Sydney, Australia PMC6944509 31909397

[40] Gvan de Ven GM, Tolias AS Three scenarios for continual learning. CoRR abs/1904.07734 (2019) 1904.07734

[41] Yoon J, Yang E, Lee J et al (2018) Lifelong learning with dynamically expandable networks. BC, Canada, Vancouver

[42] Gurbuz MB, Dovrolis C ( 2022) Nispa: Neuro-inspired stability-plasticity adaptation for continual learning in sparse networks. In: Proceedings of the International Conference on Machine Learning (ICML), vol. 162, pp. 8157– 8174. PMLR, Baltimore, Maryland, USA

[43] Jin H, Kim E (2022) Helpful or harmful: Inter-task association in continual learning. Springer, Tel Aviv, Israel

[44] Hung C, Tu C, Wu C et al (2019) Compacting, picking and growing for unforgetting continual learning. MIT Press, Vancouver, Canada

[45] Rolnick D, Ahuja A, Schwarz J et al (2019) Experience replay for continual learning. Adv Neural Inf Proc Syst 32:350–360

[46] Wang Z, Liu L, Duan Y et al (2022) Continual learning through retrieval and imagination. AAAI Press, Vancouver, BC, Canada

[47] Li Z, Hoiem D (2018) Learning without forgetting. IEEE Trans Pattern Anal Machine Intell 40(12):2935–2947 10.1109/TPAMI.2017.2773081 29990101

[48] Tao X, Hong X, Chang X et al (2020) Few-shot class-incremental learning. IEE, Seattle, WA, USA

[49] Zhou D, Wang Q, Ye H, et al ( 2023) A model or 603 exemplars: Towards memory-efficient class-incremental learning. In: Proceedings of the 11th International Conference on Learning Representations, (ICLR). OpenReview.net, Kigali, Rwanda

[50] Krizhevsky A, Hinton G (2009) Learning multiple layers of features from tiny images

[51] Yogatama D, d’Autume CM, Connor JT, et al (2019) Learning and evaluating general linguistic intelligence. CoRR abs/1901.11373

[52] Zhao B, Xiao X, Gan G et al (2020) Maintaining discrimination and fairness in class incremental learning. IEEE, Seattle, WA, USA

[53] Wang S, Shi WW, Dong SL et al (2023) Semantic knowledge guided class-incremental learning. IEEE Trans Circuits Syst Video Technol 33(10):5921–5931

[54] Zhou DW, Wang FY, Ye HJ et al (2023) Pycil: a python toolbox for class-incremental learning. Sci China Inf Sci 66:9

